# Meta-analysis of the human upper respiratory tract microbiome reveals robust taxonomic associations with health and disease

**DOI:** 10.1186/s12915-024-01887-0

**Published:** 2024-04-23

**Authors:** Nick Quinn-Bohmann, Jose A. Freixas-Coutin, Jin Seo, Ruth Simmons, Christian Diener, Sean M. Gibbons

**Affiliations:** 1 Institute for Systems Biology, Seattle, WA 98109 USA; 2 Molecular Engineering Graduate Program, University of Washington, Seattle, WA 98195 USA; 3 Reckitt Health US LLC, 1 Philips Pkwy, Montvale, NJ 07645 USA; 4 Reckitt Benckiser Healthcare Ltd, 105 Bath Road, Slough, Berkshire SL1 3UH UK; 5 Department of Bioengineering, University of Washington, Seattle, WA 98195 USA; 6 Department of Genome Sciences, University of Washington, Seattle, WA 98195 USA; 7 eScience Institute, University of Washington, Seattle, WA 98195 USA

**Keywords:** Microbiome, Upper respiratory tract, Respiratory disease, Case–control, Meta-analysis, Microbiology, 16S

## Abstract

**Background:**

The human upper respiratory tract (URT) microbiome, like the gut microbiome, varies across individuals and between health and disease states. However, study-to-study heterogeneity in reported case–control results has made the identification of consistent and generalizable URT-disease associations difficult.

**Results:**

In order to address this issue, we assembled 26 independent 16S rRNA gene amplicon sequencing data sets from case–control URT studies, with approximately 2–3 studies per respiratory condition and ten distinct conditions covering common chronic and acute respiratory diseases. We leveraged the healthy control data across studies to investigate URT associations with age, sex, and geographic location, in order to isolate these associations from health and disease states.

**Conclusions:**

We found several robust genus-level associations, across multiple independent studies, with either health or disease status. We identified disease associations specific to a particular respiratory condition and associations general to all conditions. Ultimately, we reveal robust associations between the URT microbiome, health, and disease, which hold across multiple studies and can help guide follow-up work on potential URT microbiome diagnostics and therapeutics.

**Supplementary Information:**

The online version contains supplementary material available at 10.1186/s12915-024-01887-0.

## Background

The human respiratory system is a complex structure, divided into the upper respiratory tract (URT) and the lower respiratory tract (LRT), and is primarily responsible for the exchange of oxygen and carbon dioxide with the atmosphere [1]. The upper respiratory tract, with an approximate surface area of 70 m^2^, is known to harbor a diverse microbial community [2]. Beginning at birth, colonization by microbes occurs through constant exposure to the surrounding environment via aspiration, inhalation, and direct contact [2–4]. A quasi-stable community develops over time, typically consisting of genera such as *Corynebacterium* and *Dolosigranulum* in young healthy children [5] and *Corynebacterium* and *Staphylococcus* in healthy adults [6]*.* The URT, consisting of the nares, nasal passages, mouth, sinuses, pharynx, and larynx, is the section of the respiratory tract most exposed to the environment and harbors the highest bacterial density [2]. Upsetting the balance of the URT microbiome may lead to opportunistic pathogen invasion and serious respiratory tract-related disease and infection [7, 8]. Chronic respiratory diseases represent the largest disease burden worldwide, affecting over half a billion people in 2017 [9]. Pneumonia, an infection of the lungs, is a leading cause of mortality across the world, responsible for an estimated 3.2 million deaths in 2015 [10]. The likelihood of being infected by the influenza virus, another common respiratory pathogen that has caused recurrent epidemics over the past century, has been shown to be partially dependent on the composition of the URT microbiome [7, 11]. Additional respiratory conditions, such as RSV, rhinosinusitis, and recurrent respiratory allergies, have all been linked with the disruption of the URT microbiome [12–14].

Maintaining a diverse commensal microbiome can be protective against the invasion of opportunistic pathogens [2, 15]. Commensal bacteria can help to saturate metabolic niche space, preventing invasion and engraftment of potential pathogens [8]. Additionally, commensals have been shown to directly suppress viral infections through the activation of host immune responses [16]. Early exposure to certain commensal microbes can even lead to long-term immunomodulation, preventing autoimmune diseases and promoting tolerance to allergens [17, 18]. Overall, the symbiotic relationship between the URT microbiome and the host appears critical for the maintenance of human health [2, 19].

As with the gut microbiome, variability exists in the microbial composition of these URT communities across individuals. In addition to inter-individual heterogeneity and disease status, URT microbiome profiles may be shaped by other covariates known to impact community structure, such as age [1, 7], and possibly others such as technical variation (e.g., sequencing methodologies), demographics, geographic location, and sex, although these associations are not well defined. Certain keystone or core taxa are well known to have a generally positive association with health, including the genera *Dolosigranulum* and *Corynebacterium* [20–22]. The sinonasal area is predominantly colonized by *Corynebacterium* and *Staphylococcus *[23, 24], whereas the throat and tonsil areas are mostly colonized by *Streptococcus*, *Fusobacterium*, and *Prevotella* [25, 26]. Certain species in the genera *Streptococcus*, *Haemophilus*, and *Pseudomonas* have been linked to negative health outcomes and disease [1, 20, 27–29]. However, respiratory illnesses are often polymicrobial, caused or facilitated by the presence of multiple organisms [30]. Identifying consistent signatures of URT health and disease has been hampered by the variability in reported results from individual case–control studies.

Here, we conducted a meta-analysis of the composition of the URT microbiome across health and disease states to identify consistent patterns that persist across independent studies in demographically and geographically divergent cohorts within and across multiple respiratory conditions. Using 16S rRNA amplicon sequencing data collected from the nasopharynx or the oropharynx across cases and controls from 26 independent studies representing 10 respiratory diseases and conditions, we observe robust associations between the relative abundance of specific genera and disease status. The diseases, conditions, or set of conditions included in the meta-analysis are as follows: asthma [31–33], chronic obstructive pulmonary disease (COPD) [34], COVID-19 [35–37], influenza [38–40], pneumonia [41–43], respiratory allergies [44, 45], rhinosinusitis [46–48], respiratory syncytial virus (RSV, includes a range of conditions caused by the human respiratory syncytial virus) [49–51], respiratory tract infection (RTI, defined as a viral or bacterial infection of the upper or lower respiratory tract, including bronchitis) [52–54], and tonsillitis [55, 56]. Knowledge of these consistent within- or across-disease associations may help guide the development of diagnostic tools and therapeutic interventions aimed at prevention or treatment of respiratory conditions.

## Results

### Assembling case–control studies for a URT meta-analysis

To investigate the associations between the composition of the URT microbiome and disease susceptibility, we analyzed data collected from 26 independent case–control studies including 4706 total samples (study inclusion criteria outlined in the “Methods” section). Studies included in this meta-analysis had, at a minimum, publicly available 16S rRNA amplicon sequencing data and associated metadata on disease status, URT sampling site, sequencing method, and 16S rRNA hypervariable region used for amplicon sequencing. Unfortunately, additional metadata, such as age, gender, and other demographic data, were not uniformly available across all studies. Four studies included samples from both the nasopharynx and oropharynx; these samples were analyzed separately. For each study, raw data in FASTQ format were downloaded and processed through the same bioinformatic pipeline, defined in the “Methods” section below. All analyses were conducted at the genus level, given the phylogenetic resolution of partial 16S rRNA amplicon sequencing [57]. Details on each study included in this meta-analysis can be found in Additional file 1: Table 1.

### Alpha- and beta-diversity analyses show community-wide impacts of disease conditions

**Fig. 1:**
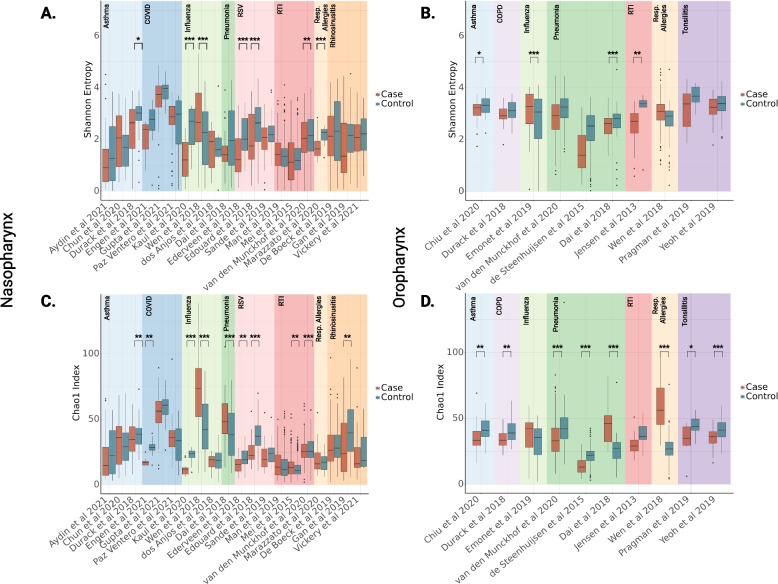
Alpha-diversity between disease cases and healthy controls for each respiratory condition. Alpha-diversity (Shannon Index or Chao1 index) is shown between cases and controls for each study. Shannon diversity for both samples from the nasopharynx (*N* = 3223) (**A**) and the oropharynx (*N* = 1313) (**B**) was calculated, as well as Chao1 richness for the nasopharynx (*N* = 3223) (**C**) and the oropharynx (*N* = 1313) (**D**). Significant differences between cases and controls were determined by independent Student’s *t*-test, two-tailed *p*-value * = *p* < 0.05, ** = *p* < 0.01, *** = *p* < 0.001

We compared URT microbiome alpha-diversity (Shannon index and Chao1 index) between disease cases and healthy controls at a per-study level. Prior to calculating diversity metrics, rarefaction to a sampling depth of 2000 reads was conducted. After rarefaction, 4536 samples remained, representing a loss of 170 samples. Due to the large compositional differences observed between the nasopharynx and oropharynx [40], diversity was investigated separately between these environments (Fig. 1A, B). Across 20 studies sampling the nasopharynx, 7 showed significant differences in alpha-diversity as measured by the Shannon Index between cases and controls, spanning asthma, influenza, RSV, RTI, and respiratory allergies (two-tailed independent Student’s *t*-test, *p* < 0.05). All but one (Wen et al., Influenza) of these significant relationships showed significantly higher alpha-diversity in healthy vs unhealthy samples (Fig. 1A). Across 10 studies sampling the oropharynx, four significant differences were observed between healthy and disease groups, for asthma, influenza, pneumonia, and RTI (two-tailed independent Student’s *t*-test, *p* < 0.05). Again, all but one (Wen et al., Influenza) showed significantly higher alpha-diversity in healthy vs unhealthy samples (Fig. 1B). Similar relationships were observed when examining taxonomic richness (Chao1 index). Among studies sampling the nasopharyngeal microbiome, 10 of 20 showed significant differences between cases and controls, including six that were also significantly enriched in the same direction in the Shannon index (Fig. 1C, two-tailed independent Student’s *t*-test, *p* < 0.05). For oropharyngeal samples, 8 of 10 studies showed significant enrichment between cases and controls (Fig. 1D, two-tailed independent Student’s *t*-test, *p* < 0.05). It has not been well established whether or not alpha-diversity of the URT microbiome is associated with disease [58]. These results indicate that changes in alpha-diversity of the URT microbiome during respiratory disease are disease-specific, not wholly consistent across studies, and lean toward an overall decline in diversity in the disease state.

**Fig. 2:**
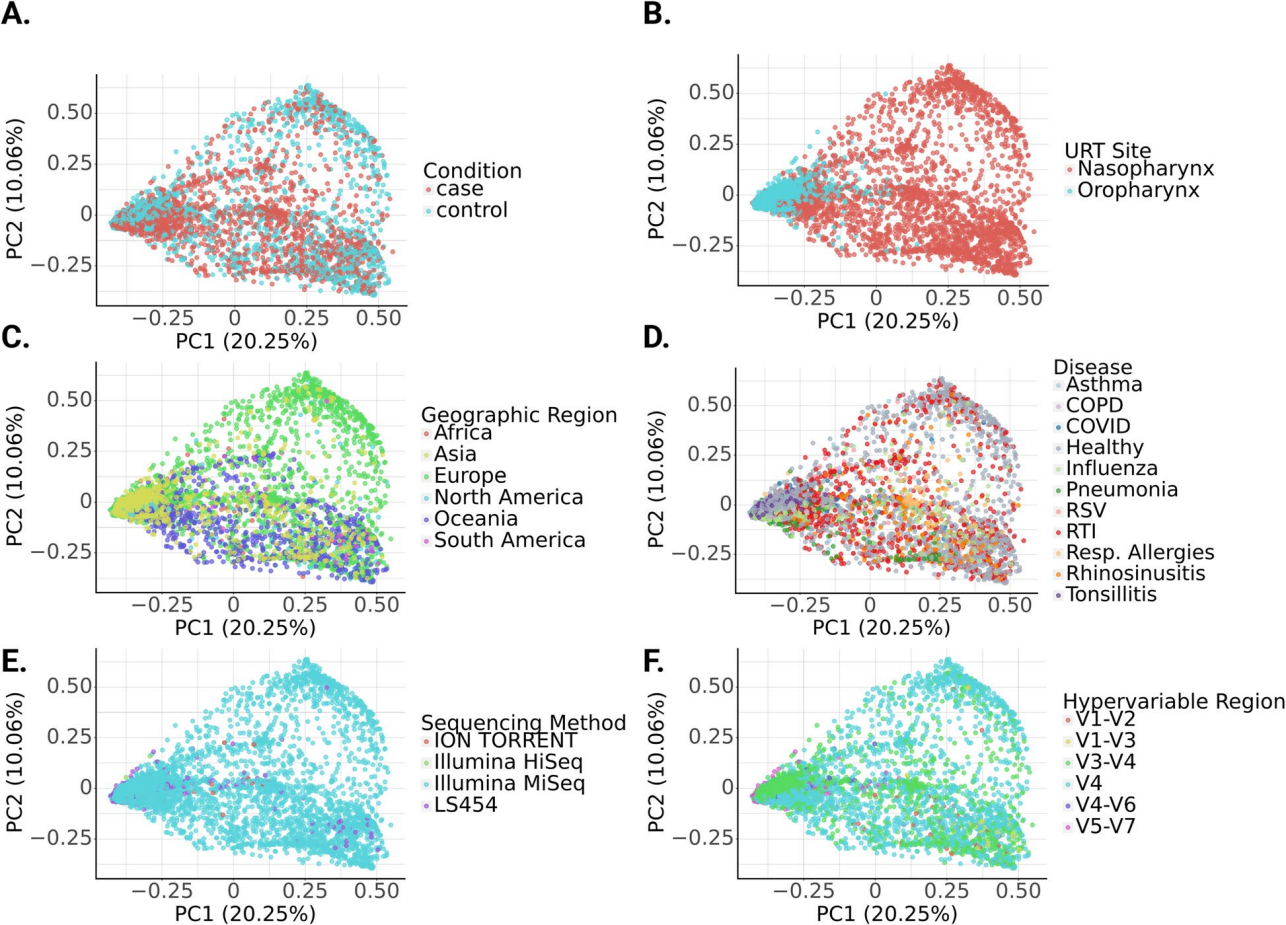
Principal coordinate analysis (PCoA) plots of genus-level Bray–Curtis distances along the first two principal coordinatess across all samples. Within subplots, each point represents a single sample (*N* = 4536). Beta-diversity was significantly associated with disease status (**A**), URT sampling site (**B**), geographic region (**C**), disease type (**D**), sequencing method (**E**), and 16S rRNA hypervariable region used for amplicon sequencing (**F**). Significant differences in beta-diversity were observed for all six parameters, as determined by PERMANOVA, *p* < 0.001 in all cases

We calculated Bray–Curtis distances at the genus level, to investigate beta-diversity patterns across studies (Fig. 2). For these analyses, all samples from all studies were pooled after rarefaction, including samples from both URT sampling sites. Analysis by PERMANOVA showed significant differences in beta-diversity between samples collected from two different URT sites, the nasopharynx and the oropharynx (Fig. 2A, PERMANOVA *p* < 0.05). This is consistent with findings that the nasopharyngeal and oropharyngeal microbiomes are compositionally distinct [59]. Additionally, a significant difference was observed between samples taken from different continents, which pushes against prior assertions that the URT microbiome is generally consistent across geographic regions [60] (Fig. 2C, PERMANOVA *p* < 0.05). As expected, significant differences were observed in Bray–Curtis dissimilarity in cases relative to controls, as well as between disease conditions (Fig. 2B, D, PERMANOVA *p* < 0.05). Finally, significant differences in beta-diversity were observed between sequencing methods, and 16S rRNA hypervariable region used for amplicon sequencing (Fig. 2E, F, PERMANOVA *p* < 0.05). These results indicated that any further analysis would necessarily require consideration of these confounding variables.

### Covariates are significantly associated with URT microbiome composition

**Fig. 3:**
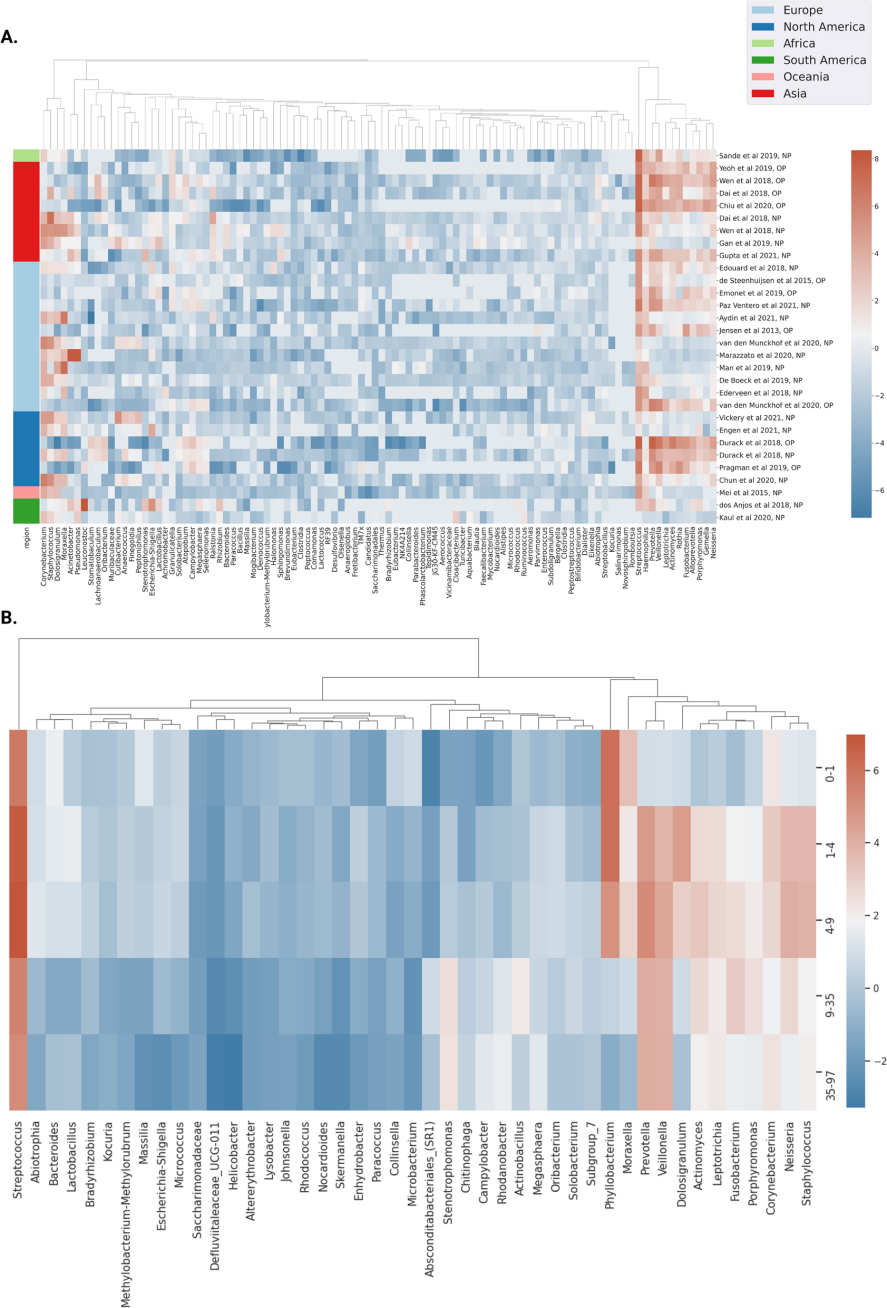
Significant differences in centered log-ratio (CLR) relative abundance of prevalent taxa between geographic regions and ages across healthy control samples. Heatmaps show significant taxonomic associations with geographic location and age in healthy controls. In both, mean CLR-transformed relative abundance is shown via color encoding, with red indicating higher CLR abundance and blue indicating lower CLR abundance. **A** Taxa displaying significant associations with geographic location in healthy controls are shown in each column (*N* = 2387). Each row represents one study, with the URT sampling site annotated (NP = nasopharynx, OP = oropharynx). Geographic region per study is shown via the color bar to the left of the heatmap. Significance was determined by multiple regression, correcting for URT sampling site, sequencing method, and 16S hypervariable region, with FDR-corrected two-tailed *p*-value < 0.05. **B** Taxa significantly associated with age are shown, for samples with available metadata for age (*N* = 554). Significance was determined by ANCOVA, treating age as a continuous variable, correcting for geographic region, URT sampling site, sequencing method, and 16S hypervariable region, with FDR-corrected two-tailed *p*-value < 0.05

Next, we aimed to examine the influence of geographic regions on taxonomic composition in healthy URT samples. Using metadata on geographic regions available for all studies, multiple regression was run for each genus to estimate the effect of geographic region (Europe, N. America, S. America, Africa, Asia, or Oceania) on centered log-ratio transformed relative abundance data, correcting for URT sampling site, sequencing method, and hypervariable region. Ninety-eight genera showed significant association with at least one geographic region (Fig. 3, multiple regression, FDR-corrected *p*-value < 0.05). FDR-corrected *p*-values and mean relative abundances of each taxon per geographic region can be found in the supplementary material (Additional file 1: Table 2).

To investigate how relative abundances of URT genera vary with age in healthy populations, ANCOVA analyses controlling for URT sampling site, geographic region, sequencing method, and 16S rRNA hypervariable region and treating age as a continuous variable were conducted. Overall, 45 genera were significantly associated with age (ANCOVA, FDR-corrected *p*-value < 0.05), based on ANCOVA containing a squared term for age to uncover potential non-linear relationships. Samples were grouped into age quantiles, in order to visualize mean CLR-transformed relative abundance across age groups for genera that showed significant associations (Fig. 3). FDR-corrected *p*-values associated with age and age^2, as well as mean relative abundances of each taxon per age quantile can be found in the supplementary material (Additional file 1: Table 3). Using a multiple regression framework similar to the age analysis (i.e., with the same set of covariates), with sex as a categorical independent variable, no genera were found to be significantly associated with sex.

### Within-study Random Forest Classifiers show how predictive URT microbiome profiles are in distinguishing cases from controls across disease types

**Fig. 4:**
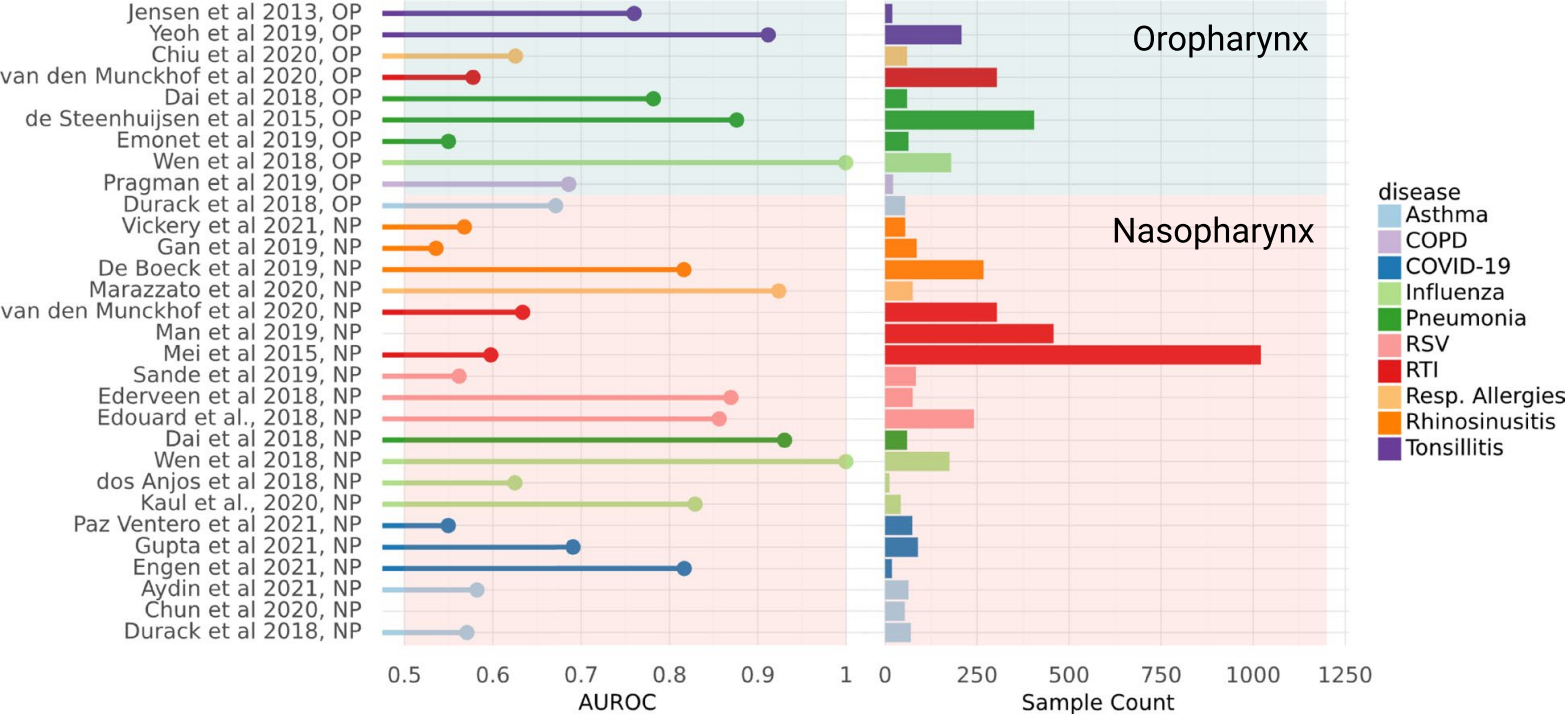
Area under the receiver-operating characteristic (AUROC) for classifying case versus control status from the URT microbiome profile across studies. AUROC values are shown for each study and sampling site (*N* = 30 data sets, 4706 samples), based on random-forest classifiers constructed using fivefold cross-validation for data from each study, separately. Values less than 0.5 are not shown. Sample count for each study is shown (range = 12–1021). Per-study disease type is shown via color encoding. Shaded background indicates the URT sampling site of each study (nasopharynx = pink; oropharynx = blue)

Random forest classifiers were constructed on a per-study basis using genus-level URT relative abundance data, with fivefold cross-validation. The capacity of these classifiers to correctly discriminate cases from controls was assessed by calculating the area under the receiver-operating characteristic (AUROC, Fig. 4) from the results of cross-validation testing. Generally, moderate classification accuracy was observed, with an average per-study AUROC of 0.71. Higher AUROC values were observed for some disease conditions, such as influenza and pneumonia. Others showed less capacity to discriminate cases from controls, such as asthma and RTI. No strong correlation was observed between study sample count (*N*) and AUROC (Pearson correlation *r* =  − 0.059, *p* = 0.75), nor between the URT sampling site and AUROC (two-tailed Student’s independent *t*-test, *t* =  − 0.76, *p* = 0.45). These results indicate that URT composition contains information that can be leveraged to predict case versus control status, but that the predictive capacity can vary substantially across diseases.

### URT microbiomes show distinct taxonomic associations across studies and disease states

**Fig. 5:**
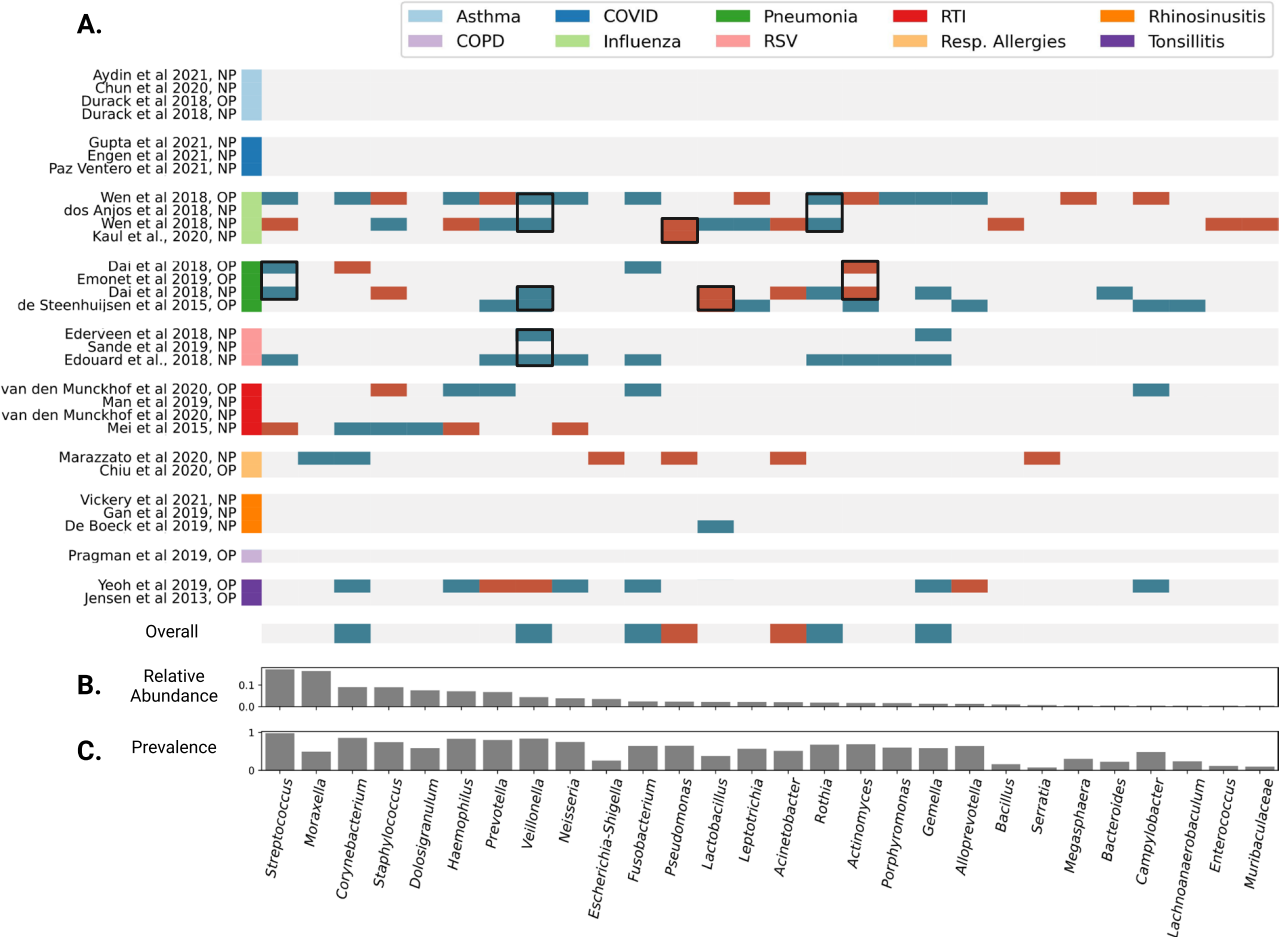
Within-study case vs. control logistic regression results at the genus-level. **A** Per-study taxonomic enrichment in cases is denoted in red, and enrichment in controls is denoted in blue (*N* = 30 data sets, 4706 samples). Blank/gray spaces indicate no significant association. Only taxa with at least one significant association are shown. Significant associations are defined as having FDR-corrected two-tailed *p*-value < 0.05. Black boxes are shown around consistent enrichments within a disease, in which taxa are enriched in the same direction in at least two studies within a disease. Overall disease associations are shown in the last heatmap row, in which enrichment in the same direction in three or more studies than in the opposite direction (*N*_same_direction_ − *N*_opposite_direction_ ≥ 3) are considered across-disease significant. **B** Mean relative abundance across all samples of each taxon shown in **A**. **C** Prevalence across all samples for each taxon shown in **A**

We next investigated whether we could identify robust taxonomic patterns of URT microbiome disruption across disease conditions. We conducted logistic regression on a per-study basis, in order to avoid cross-study comparisons due to sparsity in available covariates, with disease status as the dependent variable, iterating through separate regressions for each genus (significant genera defined as those with FDR-corrected *p* < 0.05). Studies spanning 8 disease types showed significant enrichment in at least one taxon (Fig. 5). COPD, COVID-19, and asthma were the three respiratory conditions that showed no significant taxonomic enrichments in health or disease (all FDR-corrected *p* > 0.05). Several consistent enrichments, where a taxon showed significant enrichment in the same direction in at least two studies within a disease, were observed (Fig. 5; designated by black boxes drawn around cells in the heatmap). For instance, *Pseudomonas* was consistently enriched in cases of influenza, while *Veillonella* was consistently enriched in cases of influenza, pneumonia, and RSV. Overall consistent cross-disease associations with health or disease status were defined as those genera that showed significant enrichments in the same direction in at least three more studies across all diseases than in the opposing direction (*N*_same_direction_ − *N*_opposite_direction_ ≥ 3). Following this heuristic*, Corynebacterium*, *Veillonella*, *Fusobacterium*, *Rothia*, and *Gemella* were all associated with health, although *Corynebacterium*, and *Veillonella* each showed enrichment in cases in one study. *Pseudomonas* and *Acinetobacter* were consistently associated with disease (Fig. 5). Influenza and pneumonia showed the largest number of significant enrichments among all the disease conditions analyzed. *Streptococcus* had the highest mean relative abundance of taxa with significant associations, at 17.2% ± 0.3%, followed by *Corynebacterium*, *Staphylococcus*, *Dolosigranulum*, *Haemophilis*, and *Prevotella*, all with mean relative abundances over 5% (Fig. 5). Effect sizes and FDR-corrected *p* values were recorded for each genus-disease pair (Additional file 1: Table 4).

## Discussion

The results of this meta-analysis were consistent with prior findings regarding the composition of the URT microbiome in health and disease [1] and revealed novel compositional patterns within and across diseases and between healthy individuals across age and geography. They also underscore the importance of recognizing different types of dysbioses in the URT microbiome that can potentially contribute to disease.

URT microbiome samples showed a trend toward lower alpha-diversity in disease cases, as opposed to healthy controls, in at least one study representing asthma, RTI, influenza, respiratory allergies, RSV, and pneumonia (Fig. 2A, B). Previous studies have reported similar signatures in cases of bacterial or viral infection [61, 62]. Influenza was the sole respiratory condition in which one study showed significantly higher alpha-diversity in disease cases, aligning with previous findings that alpha-diversity patterns vary depending on the disease context [43]. However, this finding will need further validation, as prior reports have found no association between URT alpha-diversity and susceptibility to influenza infection, and another study in this analysis showed an association in the opposite direction from what we report (likely due to methodological differences across analyses) [7, 63].

Bray–Curtis dissimilarity between URT communities was associated with multiple covariates: case–control status, sampling site (nasopharynx or oropharynx), disease type, geographic region, sequencing method, and 16S rRNA hypervariable region used for amplicon sequencing (Fig. 2). Concordantly, prior work has shown significant beta-diversity differences between health and disease states [61] and separation between nasopharyngeal and oropharyngeal samples, with the oropharynx harboring a more diverse microbial population than the nasopharynx [40] (Fig. 2). The significant beta-diversity differences reported here between samples from distinct geographic regions were novel. Prior work has asserted a lack of geographic signal in the URT microbiome [60]. However, it is intuitive that variation in the surrounding environment could give rise to variation in URT composition (Fig. 2). Technical differences in sequencing methodology were significantly associated with beta-diversity, as one might expect (Fig. 2). These results underscore the need to account for relevant covariates when looking for associations between URT composition and diseases that are independent of these potentially confounding factors.

We next looked into how the covariates age, sex, and geographic location shaped the taxonomic composition of the URT microbiome in healthy individuals across studies, in order to identify and isolate these signals from health and disease associations, and further indicate which covariates should be considered in future analyses (Fig. 4). Relative abundances of several taxa (*N* = 98) were observed to show significant associations with geography. *Corynebacterium*, a known health-associated taxon, showed higher mean relative abundance in samples from North America (12.0%), South America (15.2%), and Oceania (12.9%) than in samples from Africa (5.0%), Asia (4.2%), or Europe (8.6%). Conversely, *Streptococcus* showed much higher mean relative abundance in samples collected in Africa (35%) than in any other geographic region. Other taxa that show significant association with geographic region include *Gemella, Pseudomonas, Rothia*, and *Veillonella*, all of which show significant associations with health or disease via case–control analysis. Due to these significant differences in taxonomic composition, it is imperative to account for geographic location in the construction of diagnostic or therapeutic tools. Two keystone taxa, *Dolosigranulum* and *Moraxella*, were enriched in children as compared to adults, which was previously reported (Fig. 4) [5]. Additionally, we saw an increase in the health-associated taxon *Veillonella* in adults, when compared to children (Fig. 4). Due to the breadth of associations observed with age, and the purported inhibition of pathogenic invasion by some of these age-associated genera [2], we suggest that age should be included as a covariate when analyzing URT microbiome data, whenever possible. However, when age metadata are unavailable, we hope that the list of taxa provided here can be used to identify associations that may be driven by variation in age, rather than by disease. Sex showed no associations.

We ran case–control logistic regression analyses separately within each study and URT sampling site, to avoid pooling data across samples with very different demographic, biological, and methodological characteristics, similar to the approach taken in a prior meta-analysis of human gut microbiomes [64]. Robust taxonomic enrichments associated with case–control status were observed within 11 out of the 26 studies included in the meta-analysis (Fig. 5), including two studies that contained both nasopharyngeal and oropharyngeal samples. Studies from 7 of the 10 respiratory conditions included showed significant enrichment of at least one genus. Asthma, COPD, and COVID-19 were the three diseases that showed no significant URT genus-level associations, although previous URT studies have shown a microbial association with these diseases, such as with *Rothia* in COVID-19 patients [65–67].

Several consistent signatures were observed across studies within a disease. For instance, *Veillonella* was significantly enriched in controls for at least two independent studies within both pneumonia and RSV, and across OP and NP samples in the same study for influenza (Fig. 5). Two studies included in the meta-analysis, one in influenza and one in RSV, similarly report *Veillonella* enrichment in cases as compared to controls [49, 62]. Conversely, *Pseudomonas* was significantly enriched in cases across two independent studies for influenza. This association was also reported in two influenza studies included in the meta-analysis [39, 40]. *Prevotella* showed six significant enrichments across studies, but interestingly showed very inconsistent associations, with enrichment in controls in four studies and enrichment in cases in two. Here we see an example of putative dysbiosis taking many forms, and the health or disease associations of many taxa showing strong context-specificity. Across diseases, significant signatures were observed for several keystone taxa that were enriched in healthy individuals [2], like *Corynebacterium, Veillonella*, *Fusobacterium*, *Rothia*, and *Gemella*. Of these, *Corynebacterium* has been previously identified as a core taxon, putatively associated with health [1]. Additionally, these are largely abundant/prevalent taxa, with mean relative abundance above 5% for *Corynebacterium*, specifically (Fig. 5)*.* Conversely, a few genera known to harbor opportunistic pathogen species, including *Pseudomonas* and *Acinetobacter*, showed multiple associations with diseases. *Acinetobacter baumannii and Pseudomonas aeruginosa* are both known to cause disease in humans [27–29, 68]. Understanding which taxa are strongly related to health or disease, and in which contexts, will further aid the development of effective microbial diagnostics and therapeutics.

There were several limitations to our study that are important to highlight. First, there were differences in amplicon sequencing methodologies across the 26 studies included in this analysis, which introduced substantial technical biases. For example, not all studies had paired-end reads available, so we elected to use only forward reads for all studies to mitigate potential bias. Using longer, merged reads for some studies and not others would impact the efficiency of taxonomic annotation across studies (i.e., even for studies with the same variable region sequenced). Furthermore, there are often a large number of paired-end reads that fail to merge, which can lead to a substantial drop in sequencing depth in a given sample, which is another layer of bias. Additionally, samples across studies showed differences in sequencing depth. To account for this, we elected to rarefy the data to normalize sampling depth across samples. While other options exist, the current consensus in the field is that rarefaction is still optimal for comparing point estimates of alpha- and beta-diversity across samples [69].

First, while we controlled for these technical variables in our statistical testing whenever possible, incomplete metadata on these differences across studies can skew the final results. Second, many studies were missing pertinent demographic metadata, such as sex or age, which limited our statistical power by preventing us from correcting for these covariates in regressions that pooled data across all studies. It was not possible to determine whether geographic region-related trends were consistent across age groups, due to age metadata not being available for a majority of samples. Third, some studies have nearly 100-fold more samples than others, which can skew regression results if samples were pooled across studies that differed substantially in cohort size. For these reasons, the case versus control genus enrichment analyses were conducted on a per-study basis, to avoid introducing these myriad biases into the regressions. Significant case–control hits from within-study regressions that were consistent across studies provided strong support for disease-specific associations that are independent of the aforementioned limitations.

## Conclusions

Overall, these findings point to different flavors of dysbiosis that distinguish different disease states in the URT. In some cases, the disease state is characterized by a loss of putatively beneficial commensals, such as *Veillonella* in influenza, pneumonia, and RSV, and in other cases, it is characterized by the gain of putatively pathogenic taxa such as *Pseudomonas* in influenza, which mirrors what has been found across diseases in the human gut microbiome [64]. Future work should leverage these results to help guide the development of diagnostics and therapeutics for the URT.

## Methods

### Systematic review of relevant studies

A systematic review was conducted using two main search engines (PubMed and Embase) to retrieve all relevant publications describing microbiome sequencing in the human upper respiratory tract. A PRISMA flow chart (Additional file 2: Fig. S1) shows how the publications were screened, identified as relevant, and finally selected based on inclusion and exclusion criteria. Briefly, a total of 153,586 reports were identified using relevant keywords such as “microbiome,” “16S rRNA,” “URT,” “oropharynx,” “nasopharynx,” and “larynx.” Of these, 37,083 were classified as conference abstracts, conference papers, short surveys, and book chapters and therefore were excluded from the analysis. Additional exclusion criteria included 16S rRNA studies from non-human URT, which filtered out 115,883 manuscripts, leaving only 620 manuscripts. Of these 620 manuscripts, a very strict and manual pre-selection was conducted to eliminate those with irrelevant topics or disease conditions, such as studies that involved interventions or those without healthy patient controls, as well as studies with unavailable sequencing data, incomplete metadata, or duplicate manuscripts that referred to the same clinical study. This pre-selection step reduced the number of manuscripts by approximately 90%, leaving only 68 manuscripts. The final selection step was conducted manually to ensure the public availability of well-curated metadata and corresponding raw sequencing data files. This step also excluded studies from overrepresented disease conditions, so that no more than 3 studies were selected per disease condition. At the end, a total of 26 peer-reviewed publications survived all inclusion and exclusion criteria, yielding a total of 10 URT-related conditions (asthma, chronic obstructive pulmonary disease, COVID-19, influenza, pneumonia, respiratory allergies, rhinosinusitis, RSV, respiratory tract infection, tonsillitis) with 1–3 studies per condition representing a total of 4,706 samples.

### 16S rRNA amplicon sequencing URT cohorts

All phylogenetic and read count data used in this study consisted of 16S rRNA gene amplicon sequencing data, with multiple hypervariable regions sequenced across studies, spanning the V1 to V7 regions. A full list of the 26 data sets analyzed in this study, along with links to SRA accession numbers and accompanying metadata, can be found in Additional file 1: Table 1. The studies contained between 12 and 1021 subjects and varied in age from birth to 97 years old (in studies where age metadata was available), with more representation of young individuals. Studies were conducted in all six inhabited continents, with more representation from Europe and North America. 16S rRNA amplicon sequencing data consisting of FASTQ files, along with associated metadata, were downloaded from the NCBI SRA. While some studies included paired-end sequencing reads, only forward reads were used to maintain better analytical consistency across all studies and to avoid biases in the efficiency of taxonomic assignment between studies. Following data collection, all FASTQ data were imported into QIIME2 version 2022.8.3 [70] for further processing and analysis. Data were imported through the construction of a single-end Phred33v2 FASTQ manifest for each dataset. Following import, quality control and filtering in the QIIME2 DADA2 (v1.12.1) [71] plug-in removed chimeric sequences, trimmed left ends of all sequences by 10 bp to remove primers, truncated sequences uniformly at 200 bp, and identified amplicon sequence variants (ASVs). In total 623,507,314 reads were filtered, with 134,649,099 removed for poor quality or chimerism.

### Data preprocessing and taxonomic classification

The Silva high-quality rRNA gene database version 138 was used to assign taxonomy to ASVs [72]. The full-length 16S rRNA classifier was used due to heterogeneity in the hypervariable region used for sequencing between studies. Mean classification at the genus level was 86.0% (Additional file 1: Table 5; Additional file 2: Fig. S2). At the species level, classification was unsuccessful, with a mean classification of 13.9%. As a result, all subsequent analyses were conducted at the genus level by binning ASV counts together based on their genus-level annotations. All subsequent data analysis was managed using pandas (v1.4.4) in Python (v3.8.13).

### Alpha-diversity analyses

To investigate alpha-diversity, QIIME2 artifacts containing sequences for each study were merged into a single dataframe. Prior to calculation, algorithmic filtering removed any taxa with fewer than two reads per study, and any taxa present in less than 5% of samples across a study. This merged data frame was converted into a QIIME2 artifact and rarefied using the qiime feature-table rarefy function to a sampling depth of 2000. Alpha-diversity was calculated in QIIME2 via the alpha function within the diversity plugin. Shannon entropy and Chao1 index were used to estimate alpha-diversity for all samples included in the meta-analysis. Shannon entropy and Chao1 index for cases and controls within each disease were plotted and significant differences across groups were tested using two-tailed independent Student’s *t*-test (*p* < 0.05) in SciPy (v1.8.1).

### Beta-diversity analyses

To estimate beta-diversity, the filtered and rarefied genus count table constructed previously was used to construct a Bray–Curtis dissimilarity matrix using the beta function in the QIIME2 diversity plugin. Subsequently, principal coordinate analysis (PCoA) was used to analyze and visualize overall beta-diversity in scikit-bio version 0.5.7. Significant differences in beta-diversity were observed along multiple axes, including case vs. control status, disease type, geographic location, URT sampling site, sequencing method, and 16S rRNA hypervariable region as determined by PERMANOVA, using the adonis function within the diversity plugin for QIIME2.

### URT compositional patterns across geographic regions

A genus-level abundance matrix was constructed using only healthy control samples, and taxa with fewer than two reads per study or those present in fewer than 5% of samples across a study were removed. To examine the association between geographic location and centered log-ratio (CLR) transformed relative abundance of common taxa, multiple regression was used to determine significant enrichments of taxa in each geographic region while correcting for URT sampling site, sequencing method, and 16S rRNA hypervariable region using the formula “clr ~ region + v_region + sequencing + URT” in statsmodels (v0.13.5) [73]. For the purpose of these analyses, the continents in which studies took place were used as the geographic regions, as too many countries were represented to have appropriate statistical power at smaller geographic scales. As sex and age metadata were not available for 61.5% of the studies, these covariates were not accounted for in this analysis. Multiple comparison correction for *p*-values was done using the Benjamini–Hochberg method for adjusting the false discovery rate (FDR) [74], using statsmodels (v0.14.1). Per-study mean CLR-transformed relative abundance of taxa identified to be significantly enriched in at least one geographic region (multiple regression, FDR-corrected *p* < 0.05) were added to a clustered heatmap, with color encoding the average CLR-transformed relative abundances in each context. Columns containing average CLR-transformed relative abundances were clustered via an agglomerative clustering algorithm using clustermap in seaborn (v0.12.2).

### URT microbiome-age associations

Associations between age and CLR-transformed relative abundances was analyzed via ANCOVA in statsmodels. Using 10 studies for which age metadata was available, ANCOVA was conducted using the following formula “clr ~ age + age^2^ + variable_region + sequencing + URT_site + region” that was used to determine significant associations with age, accounting for URT sampling site, geographic region, sequencing method, and 16S rRNA hypervariable region. The square term for age was included to determine if non-linear relationships existed between CLR and age. The *p*-values were corrected for multiple comparisons via the Benjamini–Hochberg FDR correction as previously described. Samples were split into quantiles by age for visualization. Significantly associated taxa (FDR-corrected *p* < 0.05) were added to a heatmap with color encoding the average CLR-transformed relative abundances.

### URT microbiome associations with sex

Associations between sex and genus-level CLR abundances were determined via multiple regression. Using the 10 studies for which sex metadata was available, multiple regression were conducted using the following formula: “clr ~ sex + variable_region + sequencing + URT_site + region” in statsmodels. The resulting *p*-values were corrected for multiple comparisons via the Benjamini–Hochberg FDR correction. After correction, no taxa showed a significant association with sex.

### Supervised classification of cases and control

Random forest classifiers were constructed for each study to classify cases and controls within each study using scikit-learn (v0.24.1) [74, 75]. Classifiers were constructed with fivefold cross-validation, using the scikit-learn StratifiedKFold function to shuffle data. The RandomForestClassifier function within scikit-learn was used to construct classifiers with n_estimators = 100. Area under the curve of the receiver-operating characteristic was calculated using the results of cross-validation testing, using the cross_val_predict and roc_auc_score functions in scikit-learn*.*

### URT microbiome-disease associations

To investigate the association between genera in the URT microbiome and disease, sample read counts were normalized using a CLR transformation, as above. Logistic regressions used case–control status as the dependent variable and CLR-transformed abundance as the independent variable, following the formula “case_control_status ~ clr” in statsmodels. Regressions were run separately within each study and sampling site. By running separate analyses within each study and sampling site, key confounders like geographic location, sampling site, 16S rRNA hypervariable region, and sequencing method were constant within a given regression analysis. Mean relative abundance of each taxon within a given study and sampling site found to be significant was calculated for visualizations. *P*-values were FDR-corrected as described above. Significance was assigned to any association with an FDR-corrected *p*-value less than 0.05. Results were plotted in a binary heatmap, with significant health-associated genera designated as blue and disease-associated genera designated as red. Heatmaps were constructed using seaborn.

### Supplementary Information

**Additional file 1: Table S1.** Studies Inclusion. **Table S2.** Taxonomic Classification Percentage. **Table S3.** Enrichment associated with geographic region in healthy controls. **Table S4.** Enrichment associated with age in healthy controls. **Table S5.** Results from case v. control logistic regression.

**Additional file 2: Fig. S1.** Prisma Flowchart for Study Inclusion, An original search returned 153,586 studies. Filtering out conference abstracts, conference papers, short surveys and book chapters left 116,503 peer-reviewed publications. Additional screening removed 115,883 publications by screening for keywords “16S rRNA” and “human” and “upper respiratory” or “nasopharynx” or “oropharynx” or “larynx”, leaving 620 publications. Another phase of screening removed 552 publications for irrelevance (e.g., intervention studies or studies that lacked healthy controls), lack of sequencing data, unavailable, incomplete data/metadata, and duplicate studies reporting on the same cohort, leaving 68 publications. Of these, 42 were excluded due to overrepresentation of disease conditions in the final cohort, or problems with accessing the raw data and metadata. In the end, 26 publications remained, with 1-3 studies per disease. **Fig. S2.** Mean classification percentage for each study at each taxonomic level. Classification remained at or above 60% for all studies through the genus level. At the species level, a significant drop in classification percentage was observed.

## Data Availability

All data generated or analyzed during this study are included in this published article, its supplementary information files, and publicly available repositories. Additional supplementary data can be found in Additional file 1: Tables 1–5. All original data are available on the NCBI SRA under accession codes provided in Additional file 1: Table 1, with the exception of one study for which data is not publicly available. All intermediate data files for this analysis are available at Zenodo under DOI: 10.5281/zenodo.10962515. Analysis code can be found at the following GitHub repository: https://github.com/Gibbons-Lab/2023_URTmetaanalysis.

## Abbreviations

URT: Upper respiratory tract
NP: Nasopharynx
OP: Oropharynx
RTI: Respiratory tract infection
RSV: Respiratory syncytial virus
COPD: Chronic obstructive pulmonary disease
COVID-19: Coronavirus disease 2019
ASV: Amplicon sequence variant
FDR: False detection rates
CLR: Centered log-ratio
AUROC: Area under the receiver-operating characteristic
PCoA: Principal coordinate analysis
ANCOVA: Analysis of covariance
NCBI SRA: National Center for Biotechnology Information Sequence Read Archive

## References

[1] Kumpitsch C, Koskinen K, Schöpf V, Moissl-Eichinger C (2019). The microbiome of the upper respiratory tract in health and disease. BMC Biol.

[2] Man WH, de Steenhuijsen Piters WAA, Bogaert D (2017). The microbiota of the respiratory tract: gatekeeper to respiratory health. Nat Rev Microbiol.

[3] Lipinski JH, Moore BB, O’Dwyer DN (2020). The evolving role of the lung microbiome in pulmonary fibrosis. Am J Physiol Lung Cell Mol Physiol.

[4] Siegel SJ, Weiser JN (2015). Mechanisms of bacterial colonization of the respiratory tract. Annu Rev Microbiol.

[5] Bosch AATM, Levin E, van Houten MA, Hasrat R, Kalkman G, Biesbroek G (2016). Development of upper respiratory tract microbiota in infancy is affected by mode of delivery. EBioMedicine.

[6] Nesbitt H, Burke C, Haghi M (2021). Manipulation of the upper respiratory microbiota to reduce incidence and severity of upper respiratory viral infections: a literature review. Front Microbiol.

[7] Lee KH, Gordon A, Shedden K, Kuan G, Ng S, Balmaseda A (2019). The respiratory microbiome and susceptibility to influenza virus infection. Plos One.

[8] Clark SE (2020). Commensal bacteria in the upper respiratory tract regulate susceptibility to infection. Curr Opin Immunol.

[9] GBD Chronic Respiratory Disease Collaborators (2020). Prevalence and attributable health burden of chronic respiratory diseases, 1990–2017: a systematic analysis for the Global Burden of Disease Study 2017. Lancet Respir Med.

[10] Htun TP, Sun Y, Chua HL, Pang J (2019). Clinical features for diagnosis of pneumonia among adults in primary care setting: a systematic and meta-review. Sci Rep.

[11] Moghadami M (2017). A narrative review of influenza: a seasonal and pandemic disease. Iran J Med Sci.

[12] Rosas-Salazar C, Tang Z-Z, Shilts MH, Turi KN, Hong Q, Wiggins DA (2022). Upper respiratory tract bacterial-immune interactions during respiratory syncytial virus infection in infancy. J Allergy Clin Immunol.

[13] Schenck LP, Surette MG, Bowdish DME (2016). Composition and immunological significance of the upper respiratory tract microbiota. FEBS Lett.

[14] Psaltis AJ, Mackenzie BW, Cope EK, Ramakrishnan VR (2022). Unraveling the role of the microbiome in chronic rhinosinusitis. J Allergy Clin Immunol.

[15] de SteenhuijsenPiters WAA, Sanders EAM, Bogaert D (2015). The role of the local microbial ecosystem in respiratory health and disease. Philos Trans R Soc Lond B Biol Sci..

[16] Li N, Ma W-T, Pang M, Fan Q-L, Hua J-L (2019). The commensal microbiota and viral infection: a comprehensive review. Front Immunol.

[17] Olszak T, An D, Zeissig S, Vera MP, Richter J, Franke A (2012). Microbial exposure during early life has persistent effects on natural killer T cell function. Science.

[18] Gollwitzer ES, Saglani S, Trompette A, Yadava K, Sherburn R, McCoy KD (2014). Lung microbiota promotes tolerance to allergens in neonates via PD-L1. Nat Med.

[19] Li W, Ma ZS (2023). The upper respiratory tract microbiome network impacted by SARS-CoV-2. Microb Ecol.

[20] Pettigrew MM, Laufer AS, Gent JF, Kong Y, Fennie KP, Metlay JP (2012). Upper respiratory tract microbial communities, acute otitis media pathogens, and antibiotic use in healthy and sick children. Appl Environ Microbiol.

[21] Biesbroek G, Tsivtsivadze E, Sanders EAM, Montijn R, Veenhoven RH, Keijser BJF (2014). Early respiratory microbiota composition determines bacterial succession patterns and respiratory health in children. Am J Respir Crit Care Med.

[22] Bomar L, Brugger SD, Yost BH, Davies SS, Lemon KP (2016). Corynebacterium accolens releases antipneumococcal free fatty acids from human nostril and skin surface triacylglycerols. MBio.

[23] Kim HJ, Jo A, Jeon YJ, An S, Lee K-M, Yoon SS (2019). Nasal commensal Staphylococcus epidermidis enhances interferon-λ-dependent immunity against influenza virus. Microbiome.

[24] Menberu MA, Liu S, Cooksley C, Hayes AJ, Psaltis AJ, Wormald P-J (2021). Corynebacterium accolens has antimicrobial activity against Staphylococcus aureus and methicillin-resistant S. aureus pathogens isolated from the sinonasal niche of chronic rhinosinusitis patients. Pathogens.

[25] Zaura E, Keijser BJF, Huse SM, Crielaard W (2009). Defining the healthy “core microbiome” of oral microbial communities. BMC Microbiol.

[26] Bach LL, Ram A, Ijaz UZ, Evans TJ, Lindström J (2020). A longitudinal study of the human oropharynx microbiota over time reveals a common core and significant variations with self-reported disease. Front Microbiol.

[27] Harrison A, Mason KM, Emerging H, Infections R-E (2015). Pathogenesis of Haemophilus influenzae in humans. Hoboken.

[28] Qin S, Xiao W, Zhou C, Pu Q, Deng X, Lan L (2022). Pseudomonas aeruginosa: pathogenesis, virulence factors, antibiotic resistance, interaction with host, technology advances and emerging therapeutics. Signal Transduct Target Ther.

[29] Brouwer S, Rivera-Hernandez T, Curren BF, Harbison-Price N, De Oliveira DMP, Jespersen MG (2023). Pathogenesis, epidemiology and control of Group A Streptococcus infection. Nat Rev Microbiol.

[30] Stearns JC, Davidson CJ, McKeon S, Whelan FJ, Fontes ME, Schryvers AB (2015). Culture and molecular-based profiles show shifts in bacterial communities of the upper respiratory tract that occur with age. ISME J.

[31] Aydin M, Weisser C, Rué O, Mariadassou M, Maaß S, Behrendt A-K, et al. The rhinobiome of exacerbated wheezers and asthmatics: insights from a German pediatric exacerbation network. Front Allergy. 2021;2:667562. NCBI SRA https://www.ncbi.nlm.nih.gov/bioproject/?term=PRJNA714100 (2021) 10.3389/falgy.2021.667562 PMC8974812 35386977

[32] Chun Y, Do A, Grishina G, Grishin A, Fang G, Rose S, et al. Integrative study of the upper and lower airway microbiome and transcriptome in asthma. JCI Insight. 2020;5. NCBI SRA https://www.ncbi.nlm.nih.gov/bioproject/?term=PRJNA601757 (2020) 10.1172/jci.insight.133707 PMC7141394 32161195

[33] Durack J, Huang YJ, Nariya S, Christian LS, Ansel KM, Beigelman A, et al. Bacterial biogeography of adult airways in atopic asthma. Microbiome. 2018;6:104. NCBI SRA https://www.ncbi.nlm.nih.gov/bioproject/?term=PRJEB15534 (2016), https://www.ncbi.nlm.nih.gov/bioproject/?term=PRJEB22676 (2018) 10.1186/s40168-018-0487-3 PMC5994066 29885665

[34] Pragman AA, Knutson KA, Gould TJ, Isaacson RE, Reilly CS, Wendt CH. Chronic obstructive pulmonary disease upper airway microbiota alpha diversity is associated with exacerbation phenotype: a case-control observational study. Respir Res. 2019;20:114. NCBI SRA https://www.ncbi.nlm.nih.gov/bioproject/?term=PRJNA543785 (2019) 10.1186/s12931-019-1080-4 PMC6555967 31174538

[35] Ventero MP, Cuadrat RRC, Vidal I, Andrade BGN, Molina-Pardines C, Haro-Moreno JM, et al. Nasopharyngeal microbial communities of patients infected with SARS-CoV-2 that developed COVID-19. Front Microbiol. 2021;12:637430. NCBI SRA https://www.ncbi.nlm.nih.gov/bioproject/?term=PRJNA673585 (2020) 10.3389/fmicb.2021.637430 PMC8010661 33815323

[36] Gupta A, Karyakarte R, Joshi S, Das R, Jani K, Shouche Y, et al. Nasopharyngeal microbiome reveals the prevalence of opportunistic pathogens in SARS-CoV-2 infected individuals and their association with host types. Microbes Infect. 2022;24:104880. NCBI SRA https://www.ncbi.nlm.nih.gov/bioproject/?term=PRJNA707350 (2021) 10.1016/j.micinf.2021.104880 PMC8379005 34425246

[37] Engen PA, Naqib A, Jennings C, Green SJ, Landay A, Keshavarzian A, et al. Nasopharyngeal microbiota in SARS-CoV-2 positive and negative patients. Biol Proced Online. 2021;23:10. NCBI SRA https://www.ncbi.nlm.nih.gov/bioproject/?term=PRJNA704967 (2021) 10.1186/s12575-021-00148-6 PMC8166531 34058978

[38] Borges LGDA, Giongo A, Pereira L de M, Trindade FJ, Gregianini TS, Campos FS, et al. Comparison of the nasopharynx microbiome between influenza and non-influenza cases of severe acute respiratory infections: a pilot study. Health Sci Rep. 2018;1:e47. NCBI SRA https://www.ncbi.nlm.nih.gov/bioproject/?term=PRJNA317701 (2016) 10.1002/hsr2.47 PMC6266421 30623080

[39] Kaul D, Rathnasinghe R, Ferres M, Tan GS, Barrera A, Pickett BE, et al. Microbiome disturbance and resilience dynamics of the upper respiratory tract during influenza A virus infection. Nat Commun. 2020;11:2537. NCBI SRA https://www.ncbi.nlm.nih.gov/bioproject/?term=PRJNA240559 (2014), https://www.ncbi.nlm.nih.gov/bioproject/240562 (2014) 10.1038/s41467-020-16429-9 PMC7242466 32439901

[40] Wen Z, Xie G, Zhou Q, Qiu C, Li J, Hu Q, et al. Distinct nasopharyngeal and oropharyngeal microbiota of children with influenza A virus compared with healthy children. Biomed Res Int. 2018;2018:6362716. NCBI SRA https://www.ncbi.nlm.nih.gov/bioproject/?term=PRJNA473282 (2018), https://www.ncbi.nlm.nih.gov/bioproject/?term=PRJNA344805 (2016) 10.1155/2018/6362716 PMC6276510 30581863

[41] Dai W, Wang H, Zhou Q, Feng X, Lu Z, Li D, et al. The concordance between upper and lower respiratory microbiota in children with Mycoplasma pneumoniae pneumonia. Emerg Microbes Infect. 2018;7:92. NCBI SRA https://www.ncbi.nlm.nih.gov/bioproject/?term=PRJNA344805 (2016), https://www.ncbi.nlm.nih.gov/bioproject/?term=PRJNA431097 (2018) 10.1038/s41426-018-0097-y PMC5964150 29789582

[42] Emonet S, Lazarevic V, Leemann Refondini C, Gaïa N, Leo S, Girard M, et al. Identification of respiratory microbiota markers in ventilator-associated pneumonia. Intensive Care Med. 2019;45:1082–92. NCBI SRA https://www.ncbi.nlm.nih.gov/bioproject/?term=PRJEB20665 (2018) 10.1007/s00134-019-05660-8 PMC6667422 31209523

[43] de Steenhuijsen Piters WAA, Huijskens EGW, Wyllie AL, Biesbroek G, van den Bergh MR, Veenhoven RH, et al. Dysbiosis of upper respiratory tract microbiota in elderly pneumonia patients. ISME J. 2016;10:97–108. NCBI SRA https://www.ncbi.nlm.nih.gov/bioproject/?term=PRJNA276495 (2015) 10.1038/ismej.2015.99 PMC4681870 26151645

[44] Chiu C-Y, Chan Y-L, Tsai M-H, Wang C-J, Chiang M-H, Chiu C-C (2020). Cross-talk between airway and gut microbiome links to IgE responses to house dust mites in childhood airway allergies. Sci Rep.

[45] Marazzato M, Zicari AM, Aleandri M, Conte AL, Longhi C, Vitanza L, et al. 16S metagenomics reveals dysbiosis of nasal core microbiota in children with chronic nasal inflammation: role of adenoid hypertrophy and allergic rhinitis. Front Cell Infect Microbiol. 2020;10:458. NCBI SRA https://www.ncbi.nlm.nih.gov/bioproject/?term=PRJNA554533 (2019) 10.3389/fcimb.2020.00458 PMC7492700 32984078

[46] De Boeck I, Wittouck S, Martens K, Claes J, Jorissen M, Steelant B, et al. Anterior nares diversity and pathobionts represent sinus microbiome in chronic rhinosinusitis. mSphere. 2019;4. NCBI SRA https://www.ncbi.nlm.nih.gov/bioproject/?term=PRJEB30316 (2019), https://www.ncbi.nlm.nih.gov/bioproject/?term=PRJEB23057 (2017) 10.1128/mSphere.00532-19 PMC6881717 31776238

[47] Gan W, Yang F, Tang Y, Zhou D, Qing D, Hu J, et al. The difference in nasal bacterial microbiome diversity between chronic rhinosinusitis patients with polyps and a control population. Int Forum Allergy Rhinol. 2019;9:582–92. NCBI SRA https://www.ncbi.nlm.nih.gov/bioproject/?term=PRJNA493980 (2018) 10.1002/alr.22297 30720930

[48] Vickery TW, Armstrong M, Kofonow JM, Robertson CE, Kroehl ME, Reisdorph NA, et al. Specialized pro-resolving mediator lipidome and 16S rRNA bacterial microbiome data associated with human chronic rhinosinusitis. Data Brief. 2021;36:107023. NCBI SRA https://www.ncbi.nlm.nih.gov/bioproject/?term=PRJNA678776 (2020) 10.1016/j.dib.2021.107023 PMC8076692 33937456

[49] Edouard S, Million M, Bachar D, Dubourg G, Michelle C, Ninove L, et al. The nasopharyngeal microbiota in patients with viral respiratory tract infections is enriched in bacterial pathogens. Eur J Clin Microbiol Infect Dis. 2018;37:1725–33. NCBI SRA https://www.ncbi.nlm.nih.gov/bioproject/?term=PRJEB14780 (2018) 10.1007/s10096-018-3305-8 30033505

[50] Ederveen THA, Ferwerda G, Ahout IM, Vissers M, de Groot R, Boekhorst J, et al. Haemophilus is overrepresented in the nasopharynx of infants hospitalized with RSV infection and associated with increased viral load and enhanced mucosal CXCL8 responses. Microbiome. 2018;6:10. NCBI SRA https://www.ncbi.nlm.nih.gov/bioproject/?term=PRJEB20811 (2017) 10.1186/s40168-017-0395-y PMC5765694 29325581

[51] Sande CJ, Njunge JM, Mwongeli Ngoi J, Mutunga MN, Chege T, Gicheru ET, et al. Airway response to respiratory syncytial virus has incidental antibacterial effects. Nat Commun. 2019;10:2218. NCBI SRA https://www.ncbi.nlm.nih.gov/bioproject/?term=PRJEB28053 (2018) 10.1038/s41467-019-10222-z PMC6525170 31101811

[52] Man WH, van Houten MA, Mérelle ME, Vlieger AM, Chu MLJN, Jansen NJG, et al. Bacterial and viral respiratory tract microbiota and host characteristics in children with lower respiratory tract infections: a matched case-control study. Lancet Respir Med. 2019;7:417–26. NCBI SRA https://www.ncbi.nlm.nih.gov/bioproject/?term=PRJNA428382 (2018) 10.1016/S2213-2600(18)30449-1 PMC7172745 30885620

[53] Teo SM, Mok D, Pham K, Kusel M, Serralha M, Troy N, et al. The infant nasopharyngeal microbiome impacts severity of lower respiratory infection and risk of asthma development. Cell Host Microbe. 2015;17:704–15. NCBI SRA https://www.ncbi.nlm.nih.gov/bioproject/?term=PRJNA275918 (2015) 10.1016/j.chom.2015.03.008 PMC4433433 25865368

[54] van den Munckhof EHA, Hafkamp HC, de Kluijver J, Kuijper EJ, de Koning MNC, Quint WGV, et al. Nasal microbiota dominated by Moraxella spp. is associated with respiratory health in the elderly population: a case control study. Respir Res. 2020;21:181. NCBI SRA https://www.ncbi.nlm.nih.gov/bioproject/?term=PRJNA596902 (2019) 10.1186/s12931-020-01443-8 PMC7362441 32664929

[55] Jensen A, Fagö-Olsen H, Sørensen CH, Kilian M (2013). Molecular mapping to species level of the tonsillar crypt microbiota associated with health and recurrent tonsillitis. Plos One.

[56] Yeoh YK, Chan MH, Chen Z, Lam EWH, Wong PY, Ngai CM, et al. The human oral cavity microbiota composition during acute tonsillitis: a cross-sectional survey. BMC Oral Health. 2019;19:275. NCBI SRA https://www.ncbi.nlm.nih.gov/sra/?term=PRJNA559766 (2019) 10.1186/s12903-019-0956-5 PMC6896734 31806002

[57] Caudill MT, Brayton KA (2022). The use and limitations of the 16S rRNA sequence for species classification of anaplasma samples. Microorganisms.

[58] Avalos-Fernandez M, Alin T, Métayer C, Thiébaut R, Enaud R, Delhaes L (2022). The respiratory microbiota alpha-diversity in chronic lung diseases: first systematic review and meta-analysis. Respir Res.

[59] Lemon KP, Klepac-Ceraj V, Schiffer HK, Brodie EL, Lynch SV, Kolter R (2010). Comparative analyses of the bacterial microbiota of the human nostril and oropharynx. MBio.

[60] Gupta VK, Paul S, Dutta C (2017). Geography, ethnicity or subsistence-specific variations in human microbiome composition and diversity. Front Microbiol.

[61] Abreu NA, Nagalingam NA, Song Y, Roediger FC, Pletcher SD, Goldberg AN (2012). Sinus microbiome diversity depletion and Corynebacterium tuberculostearicum enrichment mediates rhinosinusitis. Sci Transl Med..

[62] Li J, Jing Q, Li J, Hua M, Di L, Song C (2023). Assessment of microbiota in the gut and upper respiratory tract associated with SARS-CoV-2 infection. Microbiome.

[63] Yildiz S, Mazel-Sanchez B, Kandasamy M, Manicassamy B, Schmolke M (2018). Influenza A virus infection impacts systemic microbiota dynamics and causes quantitative enteric dysbiosis. Microbiome.

[64] Duvallet C, Gibbons SM, Gurry T, Irizarry RA, Alm EJ (2017). Meta-analysis of gut microbiome studies identifies disease-specific and shared responses. Nat Commun.

[65] Losol P, Park H-S, Song W-J, Hwang Y-K, Kim S-H, Holloway JW (2022). Association of upper airway bacterial microbiota and asthma: systematic review. Asia Pac Allergy.

[66] Hakansson AP, Orihuela CJ, Bogaert D (2018). Bacterial-host interactions: physiology and pathophysiology of respiratory infection. Physiol Rev.

[67] Watson RL, de Koff EM, Bogaert D. Characterising the respiratory microbiome. Eur Respir J. 2019;53(2):1801711. 10.1183/13993003.01711-2018. 10.1183/13993003.01711-2018 30487204

[68] Asif M, Alvi IA, Rehman SU (2018). Insight into Acinetobacter baumannii: pathogenesis, global resistance, mechanisms of resistance, treatment options, and alternative modalities. Infect Drug Resist.

[69] Weiss S, Xu ZZ, Peddada S, Amir A, Bittinger K, Gonzalez A (2017). Normalization and microbial differential abundance strategies depend upon data characteristics. Microbiome.

[70] Bolyen E, Rideout JR, Dillon MR, Bokulich NA, Abnet CC, Al-Ghalith GA (2019). Reproducible, interactive, scalable and extensible microbiome data science using QIIME 2. Nat Biotechnol.

[71] Callahan BJ, McMurdie PJ, Rosen MJ, Han AW, Johnson AJA, Holmes SP (2016). DADA2: High-resolution sample inference from Illumina amplicon data. Nat Methods.

[72] Quast C, Pruesse E, Yilmaz P, Gerken J, Schweer T, Yarza P (2013). The SILVA ribosomal RNA gene database project: improved data processing and web-based tools. Nucleic Acids Res.

[73] Seabold S, Perktold J. Statsmodels: econometric and statistical modeling with python. In: Proceedings of the 9th Python in Science Conference. SciPy; 2010;57–61. 10.25080/Majora-92bf1922-011.

[74] Benjamini Y, Hochberg Y. Controlling the false discovery rate: a practical and powerful approach to multiple testing. J R Stat Soc. 1995;57:289–300.

[75] Pedregosa F, Varoquaux G, Gramfort A, Michel V, Thirion B, Grisel O, Blondel M, et al. Scikit-Learn: Machine Learning in Python. J Mach Learn Res. 2011;12(85):2825–30.

