# Supplementary material for: Meta-analysis of the human upper respiratory tract microbiome reveals robust taxonomic associations with health and disease
Source: BMC Biol. 2024 Apr 23;22:93. doi: 10.1186/s12915-024-01887-0 (PMC11040984; doi:10.1186/s12915-024-01887-0)
Supplement: Supplementary file 2 — Additional file 2: Fig. S1. Prisma Flowchart for Study Inclusion, An original search returned 153,586 studies. Filtering out conference abstracts, conference papers, short surveys and book chapters left 116,503 peer-reviewed publications. Additional screening removed 115,883 publications by screening for keywords “16S rRNA” and “human” and “upper respiratory” or “nasopharynx” or “oropharynx” or “larynx”, leaving 620 publications. Another phase of screening removed 552 publications for irrelevance (e.g., intervention studies or studies that lacked healthy controls), lack of sequencing data, unavailable, incomplete data/metadata, and duplicate studies reporting on the same cohort, leaving 68 publications. Of these, 42 were excluded due to overrepresentation of disease conditions in the final cohort, or problems with accessing the raw data and metadata. In the end, 26 publications remained, with 1-3 studies per disease. Fig. S2. Mean classification percentage for each study at each taxonomic level. Classification remained at or above 60% for all studies through the genus level. At the species level, a significant drop in classification percentage was observed. [file 12915_2024_1887_MOESM2_ESM.pdf]

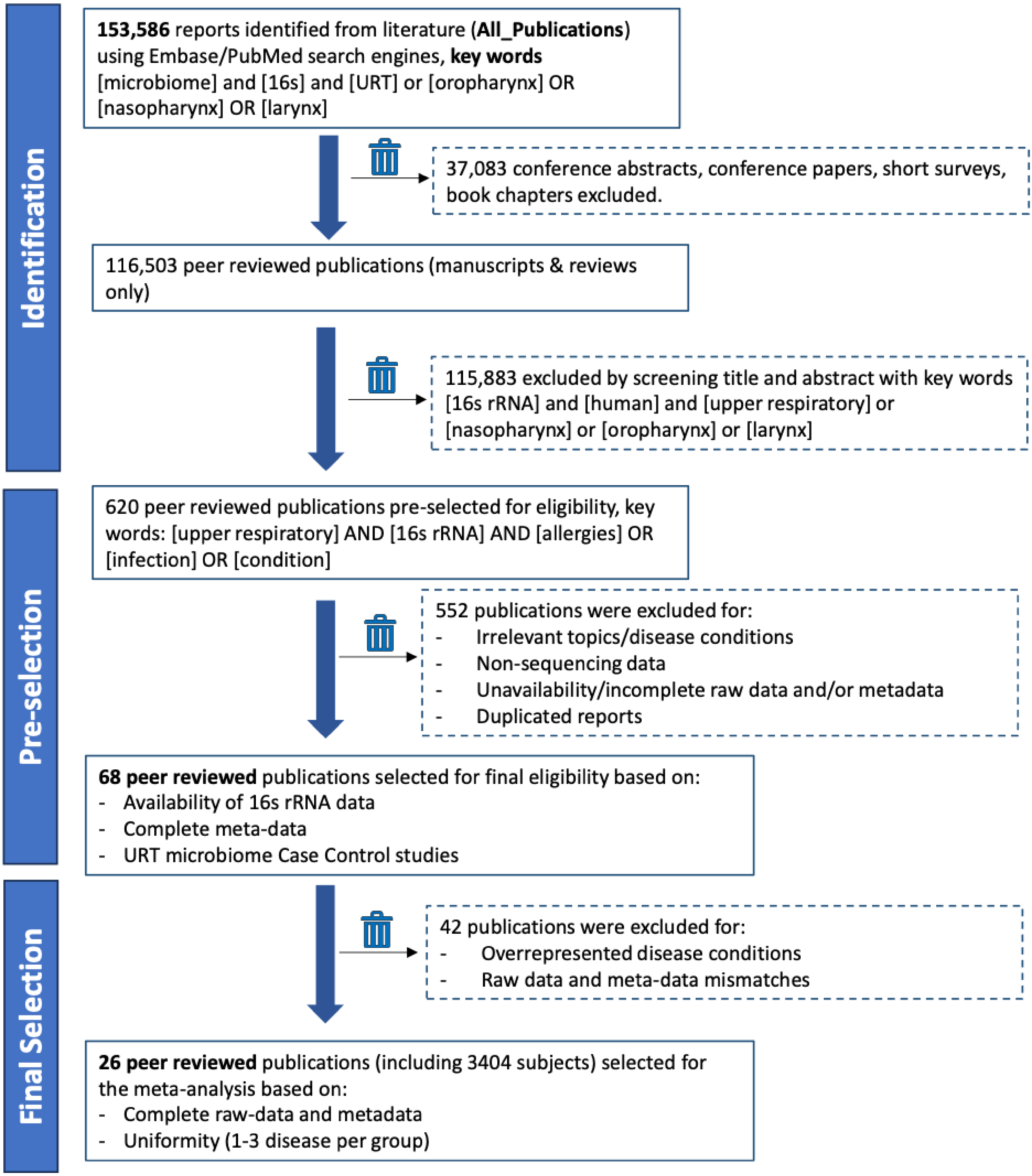
Identification
153,586 reports identified from literature (All_Publications) using Embase/PubMed search engines, key words [microbiome] and [16s] and [URT] or [oropharynx] OR [nasopharynx] OR [larynx]
37,083 conference abstracts, conference papers, short surveys, book chapters excluded.
116,503 peer reviewed publications (manuscripts & reviews only)
115,883 excluded by screening title and abstract with key words [16s rRNA] and [human] and [upper respiratory] or [nasopharynx] or [oropharynx] or [larynx]
Pre-selection
620 peer reviewed publications pre-selected for eligibility, key words: [upper respiratory] AND [16s rRNA] AND [allergies] OR [infection] OR [condition]
552 publications were excluded for:
- Irrelevant topics/disease conditions
- Non-sequencing data
- Unavailability/incomplete raw data and/or metadata
- Duplicated reports
68 peer reviewed publications selected for final eligibility based on:
- Availability of 16s rRNA data
- Complete meta-data
- URT microbiome Case Control studies
Final Selection
42 publications were excluded for:
- Overrepresented disease conditions
- Raw data and meta-data mismatches
26 peer reviewed publications (including 3404 subjects) selected for the meta-analysis based on:
- Complete raw-data and metadata
- Uniformity (1-3 disease per group)

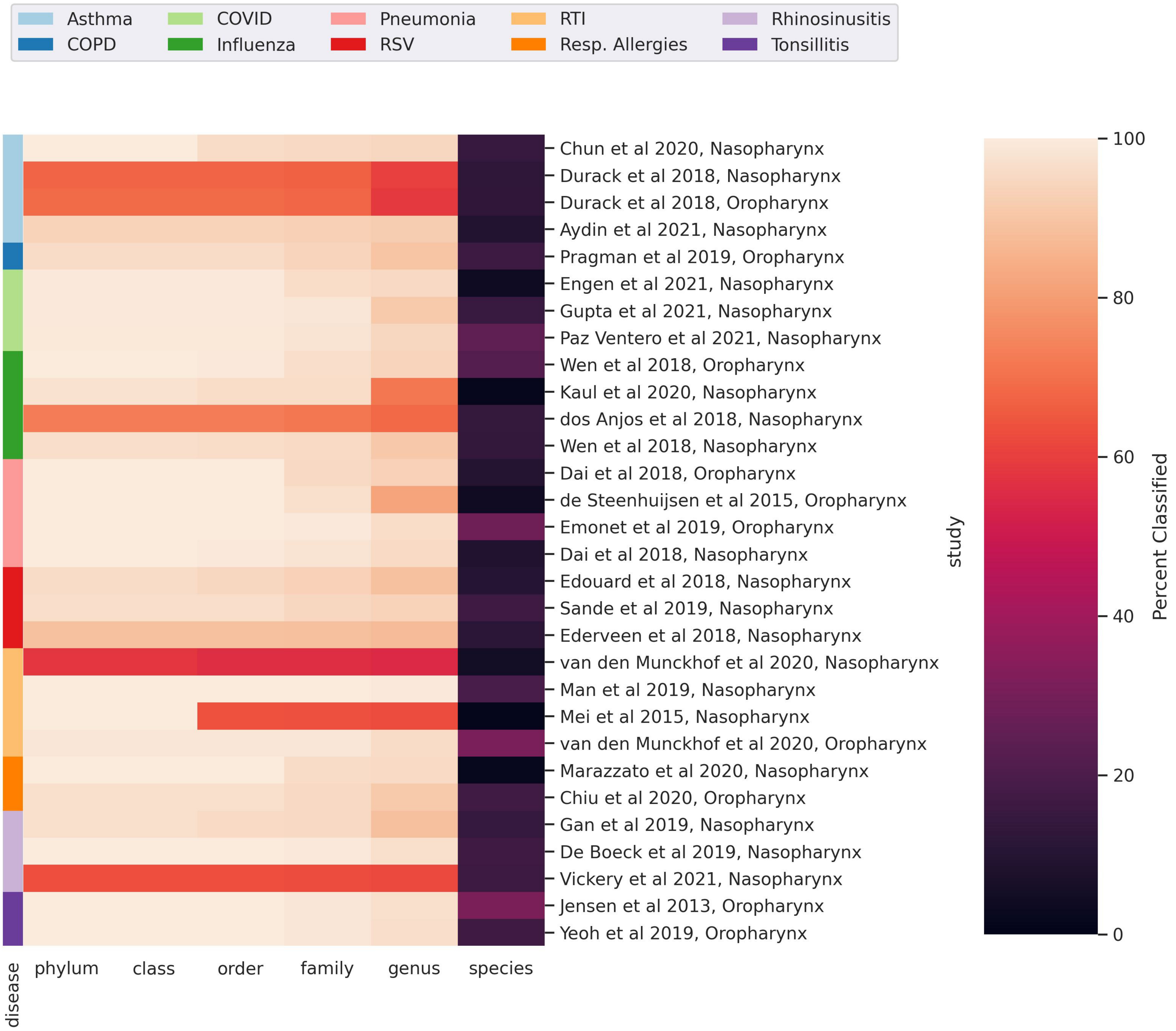
Asthma
COPD
COVID
Influenza
Pneumonia
RSV
RTI
Resp. Allergies
Rhinosinusitis
Tonsillitis
Chun et al 2020, Nasopharynx
Durack et al 2018, Nasopharynx
Durack et al 2018, Oropharynx
Aydin et al 2021, Nasopharynx
Pragman et al 2019, Oropharynx
Engen et al 2021, Nasopharynx
Gupta et al 2021, Nasopharynx
Paz Ventero et al 2021, Nasopharynx
Wen et al 2018, Oropharynx
Kaul et al 2020, Nasopharynx
dos Anjos et al 2018, Nasopharynx
Wen et al 2018, Nasopharynx
Dai et al 2018, Oropharynx
de Steenhuijsen et al 2015, Oropharynx
Emonet et al 2019, Oropharynx
Dai et al 2018, Nasopharynx
Edouard et al 2018, Nasopharynx
Sande et al 2019, Nasopharynx
Ederveen et al 2018, Nasopharynx
van den Munckhof et al 2020, Nasopharynx
Man et al 2019, Nasopharynx
Mei et al 2015, Nasopharynx
van den Munckhof et al 2020, Oropharynx
Marazzato et al 2020, Nasopharynx
Chiu et al 2020, Oropharynx
Gan et al 2019, Nasopharynx
De Boeck et al 2019, Nasopharynx
Vickery et al 2021, Nasopharynx
Jensen et al 2013, Oropharynx
Yeoh et al 2019, Oropharynx
disease
phylum
class
order
family
genus
species
study
Percent Classified
100
80
60
40
20
0
